# Determination of hazardous substances in food basket eggs in Tehran, Iran: A preliminary study

**Published:** 2015-06-15

**Authors:** Jamileh Salar-Amoli, Tahereh Ali-Esfahani

**Affiliations:** *Department of Basic Science, Faculty of Veterinary Medicine, University of Tehran, Tehran, Iran and Toxicology and animal Poisoning Research Center, University of Tehran, Tehran, Iran.*

**Keywords:** Food safety, Heavy metals, Organocholorine pesticides

## Abstract

Extensive distribution of hazardous substances in food chain and the deleterious effect of their residues on public health are a great concern of the society. Chicken eggs, as one of the most popular food commodities, in different parts of Tehran (Iran) were analyzed for two groups of hazardous substances including some organochlorine pesticides (OC) such as aldrin, lindane, dieldrin, dichlorodiphenyltrichloroethane, heptachlor and endusulfan) and heavy metals namely mercury (Hg), arsenic (As), lead (Pb), copper (Cu), cadmium (Cd), nickel (Ni) and chromium (Cr). Gas chromatography- electron capture detector, hydride-generation atomic absorption spectrometry, cold- vapor atomic absorption spectrometry and conductively coupled plasma atomic optical spectrometry were used to determine the levels of OCs, As, Hg and the others, respectively. For OCs, the results revealed none of the levels were more than maximum residues levels (MRLs), but three of them (Aldrin, lindane and endusulphan) were detectable. Moreover, 100% of 50 eggs had heavy metals with levels higher than limit of detection of the procedure. The levels of Pb and Hg in all eggs and Cd in 47% of samples were more than MRLs. It seems that the regular national monitoring of egg producing chain specially the quality of chicken feed should be taken into account seriously in order to safeguard public general health.

## Introduction

Hazardous substances, defined as persistent, bio-accumulative and toxic substances (PBTs), are chemicals which do not degrade easily in the environment. They typically accumulate in fatty tissues and are slowly metabolized often resulted in increasing their concentration through the food chain.^1^ Heavy metals such as lead (Pb), cadmium (Cd), mercury (Hg) or arsenic (As) are commonly defined as hazardous elements and cannot be broken down and persist in the environment. They therefore could enter to the food chain in many ways.^2^ In addition, other pollutants such as fat-soluble organo-chlorine (OC) pesticides such as dichlorodiphenyl-trichloroethane (DDT), lindane and endosulfan can accumulate and persist in fatty tissues. They are not easily broken down by the organisms which results in their accumulation, biomagnifications and persistence in fat tissues and deposition in food chain.^3^

Most of these hazardous substances are lipophile and accumulate in feed and food stuffs. Their chronic exposure affects several biological systems that are the major public health concerns. Endocrine disruption and cancer (e.g. breast cancer), are the most known effects of the chlorinated pesticides owing to their chronic intake.^4^^,^^5^ However, long time exposure to heavy metals such as Cd, Pb, Hg and As can cause deleterious health effects in human and animal.^7^^,^^8^

Due to the extreme nature of above mentioned substances and popularity of egg in Iranian food basket, the current study was designed to determine the levels of some hazardous heavy metals including Hg, As, Pb, Cd, copper (Cu), nickel (Ni), and chromium (Cr) according to the U.S. agency for toxic substances and disease registry (ATSDR)'s standards. Further, the contents of some OC were also assessed using Stockholm convention of persistence organic pollutants (POPs) and ATSDR (i.e. aldrin, lindane, dieldrin, DDT, heptachlor) and endusulfan, the pesticide that has recently been prohibited in Iran, in chicken eggs in some stores of different parts of Tehran (Iran).

## Materials and Methods

**Study design. **This study was conducted during summer of 2011. Fifty chickens’ eggs were collected from various batches of different local markets, (2 eggs from each market) in diverse parts of Tehran and were individually analyzed for traces of OCs (Aldrin, dieldrin, lindane, DDT, heptaclor and endosulfan) and the heavy metals (Hg, AS, Pb, Cu, Cd, Ni, and Cr).

**Determination of OC pesticides. **In order to Sample preparation and determination, all samples were prepared according to international association of analysis chemists (AOAC).^9^ each whole egg commodity was homogenized and mixed thoroughly. A representative portion of the samples (10 g) was gently blended with acetonitrile (20 mL) and sodium chloride (2 g) and cleaned up due solid phase extraction using octadecyle (C18)-bonded porous silica cartridge, all purchased from Merck Co. (Darmstadt, Germany). The levels of OCs in final solutions were measured using gas chromatograph (Model CP3800; Varian Inc., Walnut Creek, USA) equipped with a 63Ni electron capture detector. The OC pesticides mixed standard solution (1000 ppb in methanol) were purchased from Dr. Ehrenstorfer GmbH (Augsburg, Germany) to calibrate curve.

**Heavy metals (or elements). **To determine the levels of Hg, all samples were prepared according to a modified method of Segade and Tyson.^10^ Typically, 1 g of homogenized egg was introduced to 5 mL of H_2_SO_4_ and 2 mL HNO_3_ and placed in a steam bath for 10 to 20 min at 90 ^°^C to digest the sample. Then, 20 mL KMnO_4_ (5%) was pipetted to the flask to restrict probable interferences and/or discoloration of the digested solution. The digestion step was continued in the steam bath until the whole-foam was disappeared. At the next step, 5 mL hydroxyl ammonium chloride 10% as a reductant and 0.5 mL 1-octanol as a modifier were added to the solution. Finally, the contents of dishes were diluted with de-ionized water in 25 mL volumetric flask.^10^ The contents of Hg were evaluated using a cold-vapor atomic absorption spectro-meter (Model GBC 906; GBC Scientific Equipment, Melbourne, Australia) with a below limit of detection (LOD) of 0.002 ppm. An analytical standard solution (1000 ppm) and all other solutions were supplied from all purchased from Merck Co. (Darmstadt, Germany).

To determine the levels of As, a representative 4 g sample were introduced to 10 mL HCL (2M) and subsequently digested in water bath at 80 ˚C for 16 to 24 hr. In an additional stage, the samples were cooled and filtered in volumetric flask. Afterward, 200 mg kg^-1 ^potassium iodide (KI) and HCL (2 M) were mixed to the solution. After adding 0.5 mL 1- octanol to the flask, it was warmed up to 80 ˚C in a water bath for 5 to 8 min. Finally, the solutions were diluted in 25 mL volumetric flasks. Moreover, the contents of As were assessed using a hydride generation spectrometers (Model 906; GBC Scientific Equipment, Melbourne, Australia) with a LOD of 0.002 ppm. An analytical standard solution of As (1000 ppm) and all other solutions were supplied from Merck Co. (Darmstadt, Germany).

To determine the levels of Pb, Cd, Cu, Ni and Cr, 5 g of homogenized egg were transferred into evaporating dishes and placed in an oven to dry slowly and then ashed for 5 to 8 hr at 500 ˚C. The ashes were transferred into a 25 mL volumetric flasks with 4.2 mL concentrated HCL and di-ionized water. The levels of heavy metals including Pb, Cd, Cu, Ni and Cr were determined using ion conductivity plasma instrument (Model Optima™ 7300 DV; Perkin Elmer Inc. Shelton, USA) with a LOD of 0.031 ppm for Pb, 0.001 ppm for Cd and Ni, 0.004 ppm for Cu and 0.002 ppm for Cr. Analytical standard solution of mixed metals (1000 ppm) and all other solutions were supplied from Merck Co. (Darmstadt, Germany).

**Statistical Analysis. **The results were analyzed in terms of the exact levels of contaminants, their percentage in comparison to detectable levels/ LOD and also with extraneous maximum residue limit (EMRL)/maximum residue limits (MRL) of Codex Alimentarious Commission.

## Results

**Organochlorine compounds.** According to this investigation, although 3, 13 and 27% of 50 eggs samples contained detectable levels of aldrin, lindane and endusulfan, respectively (Table 1), their levels were  lower  than MRL of Codex Alimentarious Commission.^11^ Mean-while, the levels of three others (Dieldrin, DDT, and heptachlor) were less than the LOD in the present study.

**Table 1:** Levels of organochlorine pesticides and heavy metals residues in food basket eggs (n = 50) in Tehran, Iran.

**Substances**	**EMRL / MRL **		**Mean **	**Range **	**SD**	**LOD **	**> LOD (%)**	**> MRL (%)**
***Pesticide (µg kg*** ^-1^ ***)***								
**Aldrin**	100		0.030	LOD - 0.120	0.029	0.010	27	0
**Lindane**	10		0.027	< LOD - 0.060	0.007	0.010	3	0
**Deldrin**	100		0.015	< LOD	0.000	0.010	0	0
**DDT**	100		0.050	< LOD	0.000	0.010	0	0
**Endosulfan**	30		0.023	< LOD - 0.110	0.022	0.010	13	0
**Heptachlor**	50		0.020	< LOD	0.000	0.010	0	0
***Heavy metals (mg kg*** ^-1^ ***)***								
**Pb**	0.100		0.350	0.140 - 1.040	0.190	0.031	100	100
**Cd**	0.010		0.130	0.000 - 2.900	0.530	0.001	100	47
**As**	0.010		0.008	0.006 - 0.011	0.003	0.002	100	0
**Hg**	0.030		0.070	0.033 - 0.224	0.045	0.002	100	100
**Cu**	-		3.130	1.110 - 9.510	2.060	0.004	100	-
**Ni**	-		0.450	0.140 - 1.710	0.380	0.001	100	-
**Cr**	-		0.240	0.090 - 0.540	0.120	0.002	100	-

EMRL = Extraneous maximum residue level; < LOD = Below limit of detection; SD = Standard deviation; MRL = Maximum residue level.

**Table 2:** Comparison of heavy metals’ concentration (ppm) in eggs in different countries.

**Heavy metals**	**Iran**	**Pakistan** ^18^	**China** ^24^	**France** ^25^	**Nigeria** ^26^	**Belgium** ^15^	**England** ^27^	**Malaysia** ^28^
**Pb**	0.350	0.520 - 0.630	0.052	0.011	0.520 - 0.620 (0.590)	0.019 - 0.240 (0.069)	0.003	0.420
**Cd**	0.130	0.070 - 0.080	0.002	0.000	0.070 - 0.080 (0.070)	0.000 - 0.001 (0.000)	0.000	0.054
**As**	0.008	-	-	0.008	-	0.009 - 0.024 (0.016)	0.000	0.300
**Hg**	0.070	-	0.000	0.004	-	0.000 - 0.006 (0.002)	0.001	-
**Cu**	3.130	0.740 - 0.820	1.608	0.590	0.740 - 0.810 (0.780)	0.430 - 0.829 (0.603)	0.620	-
**Ni**	0.450	0.020 - 0.030	-	-	0.020 - 0.030 (0.030)	0.014 - 0.085 (0.037)	-	1.110
**Cr**	0.240	0.580 - 0.850	-	-	-	-	-	3.240

EMRL = Extraneous maximum residue level; < LOD = Below limit of detection; SD = Standard deviation; MRL = Maximum residue level.

## Discussion

Organochlorine pesticides and heavy metals are categorized as unavoidable contaminants.^12^ In spite of their banned or restricted use in many countries, their persistence properties, still make them public health threats through animal origin foods.^13^ In Iran, all the OC pesticides even linden and endosulphan have been recently banned. In this study, among 50 eggs analyzed for OC, just levels of three OC including lindane, endosulphan and aldrin were more than LOD that were less than their MRL in egg. The presence of these three pesticides in eggs may be related to their illegal usages in agriculture and reflects their delayed prohibition. The values of OC residues in Tehran were lower than in some countries, for example Jordan.^14^ In a study in Belgium, a higher incidence was reported for DDT contamination. It was detected in 90% of egg samples,^15^ while we have found the maximum residue in 27% of eggs. Tao *et al*.  have suggested that the accumulation of OCs in eggs can be accompanied with their accumulation in other tissues of chicken e.g. liver.^16^ Unlike the OCs  contamination in eggs, the levels of all heavy metals in all samples were more than the LOD.. The results revealed that 100% of samples for Pb and Hg and 47% for Cd were higher than MRL. The ATSDR in cooperation with the U.S. environmental protection agency, have introduced these three elements along with the top hazardous substances.^17^ Although the average levels of these residues in Iran are more than European committee, they are close to countries with similar industrial situation such as Pakistan, India^18^ and Turkey.^19^ The levels of all heavy metals in these countries (except Cd), are higher than the results of our study but the levels of Cd in eggs were more than Belgium ^15^ or some other countries in European Union.^20^

Most investigations have found that eggs could more likely be contaminated through oral intake of  contaminated feed by chickens.^21^^, ^^22^ However, it has been reported that indoor feeding of home reared hen lowered the level of heavy metals contamination in eggs.^23^ Poultry exposure to high levels of metals is predictive, because the main part of poultry feeding watering is based on mineral and agriculture products and also by wells water.

In conclusion, with regards to presence of OCs and heavy metals in eggs, the necessity of vigorous regular national monitoring of eggs contamination as well as quality of safe animal feed as a main source of contamination should be emphasized.
